# Loss of sclerostin promotes osteoarthritis in mice via β-catenin-dependent and -independent Wnt pathways

**DOI:** 10.1186/s13075-015-0540-6

**Published:** 2015-02-06

**Authors:** Wafa Bouaziz, Thomas Funck-Brentano, Hilène Lin, Caroline Marty, Hang-Korng Ea, Eric Hay, Martine Cohen-Solal

**Affiliations:** Inserm U1132 and university Paris 7, hopital Lariboisiere, 2 rue Ambroise Pare, 75010 Paris, France

## Abstract

**Introduction:**

Sclerostin is a Wnt inhibitor produced by osteocytes that regulates bone formation. Because bone tissue contributes to the development of osteoarthritis (OA), we investigated the role of sclerostin in bone and cartilage in a joint instability model in mice.

**Methods:**

Ten-week-old SOST-knockout (SOST-KO) and wild-type (WT) mice underwent destabilization of the medial meniscus (DMM). We measured bone volume at the medial femoral condyle and osteophyte volume and determined the OA score and expression of matrix proteins. Primary murine chondrocytes were cultured with Wnt3a and sclerostin to assess the expression of matrix proteins, proteoglycan release and glycosaminoglycan accumulation.

**Results:**

Sclerostin was expressed in calcified cartilage of WT mice with OA. In SOST-KO mice, cartilage was preserved despite high bone volume. However, SOST-KO mice with DMM had a high OA score, with increased expression of aggrecanases and type X collagen. Moreover, SOST-KO mice with OA showed disrupted anabolic–catabolic balance and cartilage damage. In primary chondrocytes, sclerostin addition abolished Wnt3a-increased expression of a disintegrin and metalloproteinase with thrombospondin motifs, matrix metalloproteinases and type X collagen by inhibiting the canonical Wnt pathway. Moreover, sclerostin inhibited Wnt-phosphorylated c-Jun N-terminal kinase (JNK) and rescued the expression of anabolic genes. Furthermore, sclerostin treatment inhibited both Wnt canonical and non-canonical JNK pathways in chondrocytes, thus preserving metabolism.

**Conclusion:**

Sclerostin may play an important role in maintaining cartilage integrity in OA.

**Electronic supplementary material:**

The online version of this article (doi:10.1186/s13075-015-0540-6) contains supplementary material, which is available to authorized users.

## Introduction

Osteoarthritis (OA), a painful joint disorder, is one of the most common chronic disabling diseases [1-3]. OA involves all joint tissues and results in cartilage breakdown [4]. Considerable progress has been made in understanding the pathophysiologic mechanisms of the cartilage loss and has pointed out the contribution of bone under mechanical conditions [5,6]. Increased subchondral bone thickness and stiffness, along with reduced mineral density of the trabecular bone beneath the cartilage, has been reported in the late stage of the disease, which suggests that bone is involved in the onset and progression of OA.

Calcified cartilage, at the interface between bone and cartilage, contains chondrocytes that might be regulated by the subchondral bone. Chondrocytes from calcified cartilage are involved in a terminal differentiation process that results in the recapitulation of endochondral ossification. Damaged calcified cartilage is associated with chondrocyte hypertrophy in OA, which releases several factors that contribute to the replacement of cartilage by bone [7,8].

The molecules of the Wnt family have been identified as major regulators of bone mass through mutations in LRP5/6 [9-11]. The Wnt pathway is inhibited in normal cartilage, but its activation promotes OA [12]. The pathway is regulated by several antagonists that inhibit canonical and/or non-canonical Wnt/β-catenin signaling.

SOST mutations in patients with high bone mass reveal sclerostin as an inhibitor of Wnt signaling [13]. Sclerostin is expressed in osteocytes and hypertrophic chondrocytes [14] and could play a regulatory role in the pathogenesis of OA. The chondrocytic expression of sclerostin was high in damaged cartilage but low in sclerotic subchondral bone [15], which suggests a possible role of sclerostin in bone and cartilage remodeling. Neither loss of SOST in aging mice nor sclerostin neutralization by systemic antibodies in rats treated with medial meniscus transection (MMT) affected articular cartilage [16]. Nevertheless, the effect of total deletion of sclerostin in mice with mechanical joint instability remains unknown.

We hypothesized that loss of SOST may contribute to the development of OA by regulating chondrocyte differentiation. We assessed the impact of sclerostin in joints of SOST-knockout (SOST-KO) mice with joint instability. We found that destabilization of the medial meniscus (DMM) induced higher OA scores in SOST-KO mice with a disrupted anabolic–catabolic balance. Furthermore, primary chondrocytes cultures revealed that sclerostin inhibited both Wnt canonical and non-canonical c-Jun N-terminal kinase (JNK) pathways, maintaining chondrocyte metabolism.

## Methods

### Animals

To evaluate the expression of sclerostin SOST during OA, we induced joint instability in 10-week-old male FVB mice by DMM of the right knee, with sham operations performed on the left knee as previously described [17]. FVB mice were killed at week 0 (*n* = 5), week 4 (*n* = 7), week 6 (*n* = 7) or week 9 (*n* = 7) after DMM. We then induced joint instability in 10-week-old male SOST-KO mice of a C57BL/6 strain supplied by Novartis (Basel, Switzerland) [18] (*n* = 10) and in wild-type (WT) littermates (*n* = 10) by DMM of the right knee, with sham operations performed on the left knee. These mice were killed at week 4 (*n* = 5) or week 6 (*n* = 5). The time point was chosen to enable us to quantify the expression of a disintegrin and metalloproteinase with thrombospondin motifs (ADAMTS) in the remaining cartilage. The mice were killed 6 weeks after induction of joint instability. The entire protocol was performed in accordance with the Guidelines for Animal Experimentation issued by the local Ethics Committee on Animal Care and Experimentation (Ethics Committee Lariboisière-Villemin number CEEALV/2012-02-01, Paris) and approved by the committee.

### Micro-CT scanning

The SkyScan 1172 high-resolution desktop X-ray microtomography (micro-CT) system (Bruker microCT, Kontich, Belgium) was used with voltage 70 kV, pixel size 6 μm and filter Alu 0.5 mm. We analyzed two different regions of interest of the medial femoral condyle in the coronal view: the subchondral plate and osteophytes in the medial tibial plateau. We calculated the bone volume/tissue volume ratio (BV/TV, %) of the subchondral plate and the TV of osteophytes by using CTan software (Bruker microCT). Three-dimensional reconstruction images were produced with CTvol (Bruker microCT). Osteophytes were measured on three-dimensional images by SkyScan 1172 micro-CT. Reconstructed images were determined while scrolling through coronal micro-CT views. Tibial osteophytes were scored at the medial surfaces using quantitative methods. Osteophytes were measured as bony spurs at the limit between the cortical bone and the subchondral bone. The region was delimited manually and expressed as TV.

### Histology

Knees were fixed in 4% paraformaldehyde for 24 hours at 4°C, then decalcified in 0.5 M ethylenediaminetetraacetic acid at 4°C for 21 days and embedded in optimum cutting temperature compound for cryosections or in paraffin. Serial 5-μm-thick sagittal sections of the medial femorotibial joints were collected at three depths at 50-μm intervals. Sections were stained with Safranin O as described elsewhere [5]. The Osteoarthritis Research Society International 2010 scoring method was used for both tibias and femurs, with a total severity score ranging from 0 to 12 [19].

Cryosections were used for sclerostin immunostaining. Sections were incubated with the mouse primary anti-sclerostin antibody (R&D Systems, Abingdon, UK). Positive cells were counted on the tibial joint cartilage surface (×25 magnification) and expressed as a percentage of total cells.

Paraffin sections were used for type X collagen and Adamts4/5 immunostaining and terminal deoxynucleotidyl transferase dUTP nick end labeling (TUNEL) assay. Slides were incubated with the antibodies mouse anti-type X collagen (Diagomics, Toulouse, France) or rabbit anti-Adamts4/5 (Abcam, Paris, France) at 4°C overnight. Negative controls were mouse (for type X collagen) or rabbit (for Adamts4/5) non-specific immunoglobulin G. The TUNEL assay was carried out according to the manufacturer’s instructions (Millipore, Molsheim, France).

### Murine cartilage harvesting and primary chondrocyte culture

Chondrocytes were harvested from 6-day-old mice as described [20], and pretreated with recombinant mouse sclerostin (R&D Systems) at 20 ng/ml for 24 h. Wnt3a-conditioned medium was added at 30% (for 15 min, 1 h, or 48 h) or interleukin 1β (IL-1β) was added at 1 ng/ml for 24 hours. This dose was determined according to dose and time effect experiments (Additional file 1: Figure S1). At 1 hour before exposure to Wnt3a-conditioned medium, SP600125 or staurosporine (Sigma-Aldrich, St Louis, MO, USA) was added at final concentrations of 5 μg/ml or 10 ng/ml, respectively. Culture media of L cells was added to the control conditions.

### β-galactosidase assay, Alcian blue staining and quantification of proteoglycan release

Chondrocytes were treated with 20 ng/ml sclerostin for 48 hours. β-galactosidase (β-Gal) activity was quantified by use of a β-Gal reporter gene kit (Roche Applied Science, Mannheim, Germany). For Alcian blue staining, chondrocytes were fixed and covered with Alcian blue at pH 0.2 (0.5% Alcian Blue 8 GX (Sigma-Aldrich) in 1 *N* HCl). Alcian blue dye was extracted with 6 M guanidine HCl for 2 hours at room temperature, then measured at 595 nm. Proteoglycan release was measured in the culture supernatant by a colorimetric method [21].

### Real time PCR

Real-time PCR involved use of SYBR Green Master Mix (Applied Biosystems, Foster City, CA, USA) in six to eight independent experiments. Averaged threshold cycle (C_t_) values were normalized to the averaged C_t_ value of *RPL13A*. Adjusted average C_t_ values were used to calculate relative expression versus the control. The following primer sequences were used:

- *ADAMTS4*: forward: 5′GGCAAGGACTATGACGC3′; reverse: 5′TCAGCCCAAGGTGAGTG3′
- *ADAMTS5*: forward: 5′TCAGCCACC ATC ACAGAA3′; reverse: 5′CCAGGGCACACCGAGTA3′
- Matrix metalloproteinase 3 (*MMP3*): forward: 5′ATGAAAATGAAGGGTCTTCCGG-3′; reverse: 5′GCAGAAGCTCCATACCAGCA-3′
- *MMP13*: forward: 5′TGATGGCACTGCTGACATCAT-3′; reverse: 5′TGTAGCCTTTGGAACTGCTT-3′
- *SOX9*: forward: 5′GAAGCTGGCAGACCAGTACC3′, reverse: 5′GGTCTCTTCTCGCTCTCGTTC3′
- Aggrecan (*ACAN*): forward: 5′CAG GGTTCCCAGTGTTCAGT3′; reverse: 5′CTGCTCCCAGTCTCAACTCC3′
- *COL2A1*: forward: 5′CCG TCATCGAGTACCGATCA3′; reverse: 5′CAGGTCAGGTCAGCCATTCA3′
- *COL10A1*: forward: 5′AAGGAGTGCCTGGACACAAT3′; reverse: 5′GTCGTAATGCTGCTGCCTAT3′
- Ribosomal protein L13 (*RPL13A*): forward: 5′GGATCCCTCCACCCTATGACA3′; reverse: 5′AGCCGAACAACCTTGAGAGC3′
- Vascular endothelial growth factor A2 (*VEGF*-A2): reverse: 5′CGGATCTTGCACAAACAAATGC3′; forward: 5′AACAAAGCCAGAAAATCACTGGA3′
- *WNT5A*: reverse: 5′GTCCTTTGAGATGGGTGGTATC3′; forward: 5′ACCTCTGGGTTAGGGAGTGTCT3′
- *WNT10B*: reverse: 5′CCACTACAGCCCAGAACCTC3′; forward: 5′GGAGAGACCCTTTCAACAACTG3′

### Western blot analysis and active Rho pull-down and detection

Whole-cell lysates were prepared, and proteins were extracted (*n* = 3 independent experiments). The following antibodies were used for incubation: anti-ADAMTS-4/5, anti-type X collagen and anti-MMP13 (Abcam), anti-CaM kinase II (CaMKII) (pan), anti-phospho-CaMKII (Thr286), anti-phospho-stress-activated protein kinase/c-Jun N-terminal kinase (SAPK/JNK) (Thr183/Tyr185) (81E11), anti-SAPK/JNK, anti-nuclear matrix protein p84 (Abcam), and anti-phospho-protein kinase C (PKC) (pan) (βII Ser660) were purchased from Cell Signaling Technology (Ozyme, Montigny-le-Bretonneux, France). Samples were treated and analyzed with the Active Rho Pull-Down and Detection kit (Pierce Biotechnology, Rockford, IL, USA).

### Statistical analysis

Data are reported as mean ± SEM. Statistical analyses were done using analysis of variance and the Mann–Whitney *U* test with StatView software (SAS Institute, Cary, NC, USA). *P* < 0.05 was set as the threshold of statistical significance.

## Results

### Expression of sclerostin is increased in calcified cartilage during the development of osteoarthritis in wild-type mice

**Figure 1:**
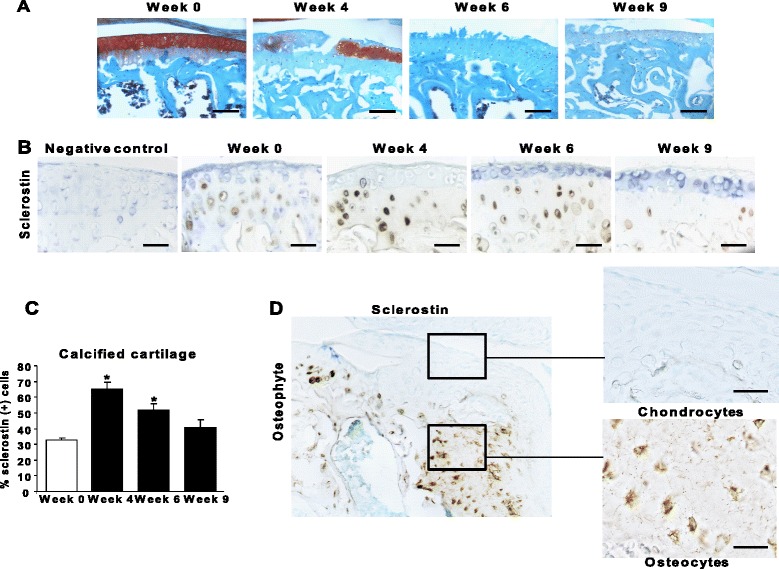
**Biphasic expression of sclerostin in calcified cartilage of mice with osteoarthritis. (A)** Safranin O stains show the progression of cartilage lesions in the tibial plateau of osteoarthritis (OA) mice. Bar, 100 nm. **(B)** Immunostaining of sclerostin in calcified cartilage is shown. Bar, 100 nm. **(C)** Percentages of sclerostin-positive cells in calcified cartilage of the tibial plateau and internal femoral condyle. **(D)** Immunostaining of sclerostin in newly formed osteophytes in the tibial plateau. Bar, 100 nm. Enlarged image bar, 40 nm. Data are mean ± SEM. *P < 0.05 versus baseline (*n* = 7 animals per time point).

In the DMM model, cartilage damage increased time-dependently in WT mice (Figure 1A). Sclerostin was expressed in late differentiated cells embedded within a calcified matrix. The proportion of sclerostin-positive cells increased from week 0 to week 4 (32.53 ± 1.32 versus 64.97 ± 4.44; *P* < 0.05), then decreased by week 6 (51.82 ± 3.89; *P* < 0.05), reaching baseline values 9 weeks after the induction of OA (40.7 ± 4.74; *P* > 0.05) (Figure 1B and C). Sclerostin was expressed in osteocytes of the subchondral bone, the expression in joint cartilage restricted to chondrocytes in the calcified zone (Figure 1B). The early upregulation indicates that sclerostin may be part of an early response to mechanical loading. We then investigated sclerostin expression in osteophytes, a hallmark of OA [22]. Sclerostin was expressed in osteophytic osteocytes, but not in chondrocytes (Figure 1D). Therefore, sclerostin was expressed only in calcified matrix-embedded cells, which suggests that it might be involved in the development of OA.

### Loss of SOST enhances subchondral bone accretion but does not affect osteophyte formation

**Figure 2:**
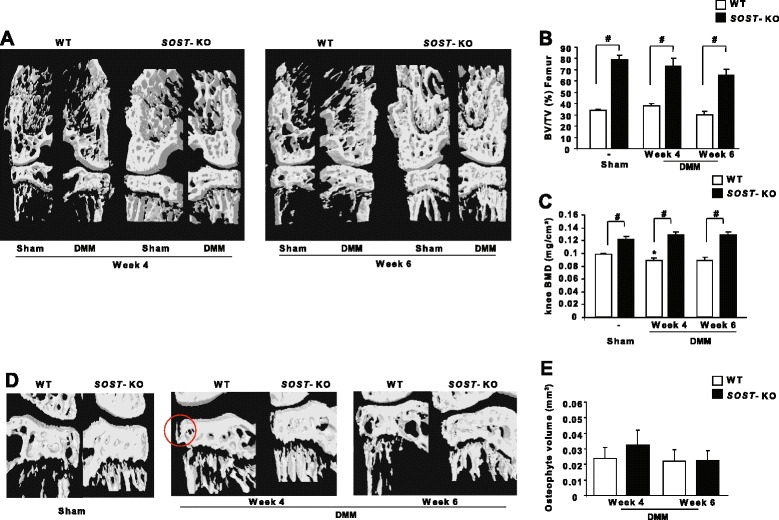
**SOST** **-knockout mice show increased bone mineral density and bone volume in subchondral and trabecular bone. (A)** Micro-CT three-dimensional coronal reconstructions of mouse knees after a sham operation (left knees) or destabilization of the medial meniscus (DMM) joint (medial femorotibial) showing subchondral bone changes. **(B)** Bone volume/tissue volume ratio (BV/TV, %) measurement in the subchondral bone of knees of wild-type (WT) and SOST-knockout (SOST-KO) mice (*n* = 5 animals at each time point). **(C)** Bone mineral density (BMD) measurement in the whole knees of WT and SOST-KO mice (*n* = 10 animals at week 0 and *n* = 5 independent animals at weeks 4 and 6 for each genotype). **(D)** Micro-CT three-dimensional coronal reconstructions of sham knees and knees with DMM showing osteophytes (red circle). **(E)** Tissue volume measurement of newly formed osteophytes from the tibial plateau of WT and SOST-KO OA mice (*n* = 5 animals at each time point). Data are mean ± SEM. #*P* < 0.05.

We evaluated BV/TV and bone mineral density (BMD) in subchondral bone of SOST-KO mice. As was previously shown [23,24], in sham-operated mice, loss of SOST promoted high bone accretion in trabecular bone and markedly increased BV/TV in the medial femoral condyle (79.36 ± 3.83 versus 34.17 ± 0.79; *P* < 0.05) (Figure 2A and B), as well as higher BMD, compared with WT mice (Figure 2C). Nevertheless, DMM did not affect BV/TV in SOST-KO mice.

In the early stages of the disease, BMD was decreased in WT mice. However, DMM did not affect subchondral BMD in SOST-KO mice (Figure 2C). Therefore, SOST may contribute to subchondral bone remodeling in OA. Osteophytes were measured at the medial tibial plateau by a method described previously [25]. No osteophyte formation was observed in the sham-operated knees, but it was present in DMM knees of WT mice. Moreover, we found no increase in osteophyte volume in SOST-KO mice compared with WT mice (*P* > 0.05) (Figure 2D and E). Thus, SOST is not involved in the regulation of osteophyte formation.

### Loss of SOST increases cartilage damage in osteoarthritis mice

**Figure 3:**
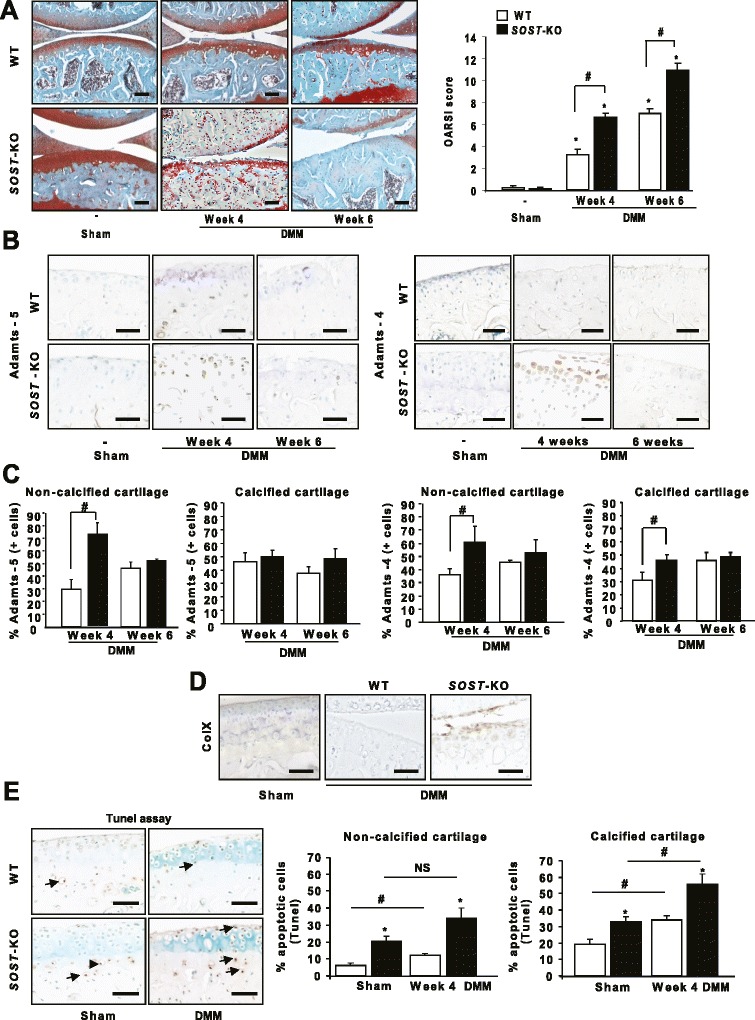
**Deletion of SOST increased damage to joint cartilage. (A)** Safranin O staining of wild-type (WT) and SOST-knockout (SOST-KO) mice after destabilization of the medial meniscus (DMM) or sham operation at weeks 4 and 6. Bar, 100 nm. Osteoarthritis (OA) score in DMM and sham-operated knees of WT and SOST-KO mice at each time point. **(B)** Immunohistochemistry of Adamts5/4 of WT and SOST-KO mice at weeks 4 and 6. Bar, 50 nm. **(C)** Quantification of information represented in (B). **(D)** Immunohistochemistry of type X collagen in WT and SOST-KO mice 4 weeks after OA induction. Bar, 50 nm. **(E)** SOST-KO increased chondrocyte apoptosis in non-calcified and calcified cartilage. Bar, 50 nm. Data are means ± SEM.*P < 0.05 compared with sham-operated mice. #*P* < 0.05 (*n* = 5 animals for each genotype).

Because the activation of Wnt signaling in chondrocytes induces cartilage damage [26], we hypothesized that sclerostin is secreted by chondrocytes or osteocytes to prevent cartilage lesions in OA. Undamaged joint cartilage was observed in both SOST-KO and WT mice at baseline (OA score: 0.16 ± 0.05 versus 0.2 ± 0.02; *P* > 0.05). In contrast, joint instability induced more severe cartilage lesions in SOST-KO than WT mice at weeks 4 and 6 (*P* < 0.05; Figure 3A). Furthermore, we analyzed the expression of Adamts4/5, two key molecules involved in cartilage degeneration during OA. Adamts5 expression was higher in SOST-KO mice in non-calcified cartilage after 4 weeks of DMM (72.16 ± 9.26 versus 29.57 ± 7.86, *P* < 0.05), but not after 6 weeks (51.4 ± 1.05 versus 46.02 ± 4.87) (Figure 3B and C). However, Adamts4 expression was increased in both non-calcified and calcified cartilage at 4 and 6 weeks after DMM (non-calcified cartilage: 60.95 ± 12.08 versus 36.04 ± 4.42; calcified cartilage 45.6 ± 4.65 versus 31.14 ± 5.67; *P* < 0.05).

In OA, overactivation of Wnt pathways stimulates the expression of hypertrophic markers and promotes apoptosis [7]. Therefore, we hypothesized that, with loss of SOST, the increased number of cartilage lesions might be associated with enhanced chondrocyte hypertrophy. Type X collagen expression was greater in DMM SOST-KO mice than in WT mice (Figure 3D), which suggests that sclerostin may inhibit the shift toward the hypertrophic phenotype. Furthermore, loss of SOST increased apoptosis in both non-calcified cartilage (sham: 16.75 ± 2.94 versus 6.18 ± 1.31; week 4 after DMM: 34.08 ± 5.76 versus 12.1 ± 1.13) and calcified cartilage (sham: 33.05 ± 3.04 versus 19.11 ± 3.22; week 4 after DMM: 55.55 ± 6.32 versus 33.76 ± 2.6) (Figure 3E). Thus, sclerostin reduces cartilage catabolism and protects chondrocytes against hypertrophy and apoptosis in OA.

### Sclerostin prevents the disruption of the anabolic/catabolic balance in chondrocytes

**Figure 4:**
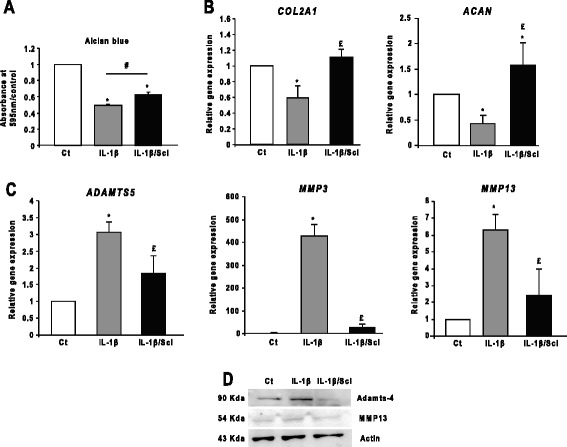
**Effect of sclerostin on chondrocyte metabolism. (A)** Alcian blue staining and spectrophotometric quantification. Sclerostin (Scl; 20 ng/ml for 24 hours) partially restored the amount of glycosaminoglycans (GAGs) released with interleukin 1β (IL-1β; 1 ng/ml for 24 hours) (*n* = 13 independent experiments). Ct, Threshold cycle. Bar, 100 nm. **(B)** and **(C)** Scl rescued the gene expression of the anabolic markers (*COL2A1* and *ACAN*), but inhibited that of catabolic markers (*ADAMTS5*, *MMP3* and *MMP13*) (*n* = 3). **(D)** Western blot analysis of Scl revealed inhibition of the increased expression of Adamts-4 and matrix metalloproteinase 13 (MMP13) with IL-1β treatment (*n* = 3). Wild-type chondrocytes were used for all the experiments. Data are mean ± SEM. *P < 0.05 compared with control; £*P* < 0.05 compared with Wnt3a; #*P* < 0.05.

To investigate whether sclerostin prevents the disruption of the anabolic–catabolic balance in chondrocytes, we activated chondrocytes with IL-1β. Sclerostin treatment partially restored the IL1-β-inhibited expression of highly sulfated glycosaminoglycans (GAGs) and anabolic markers but decreased that of catabolic markers (Figure 4).

**Figure 5:**
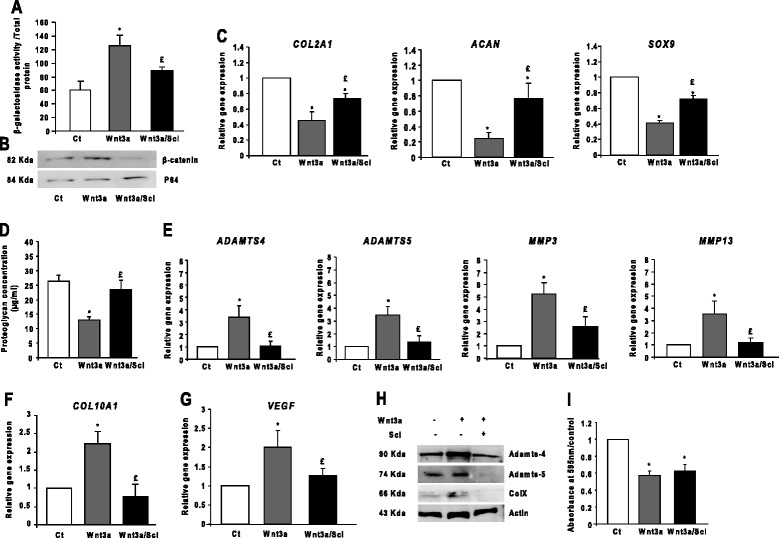
**Sclerostin preserves anabolic–catabolic balance by inhibiting chondrocyte catabolism and hypertrophy. (A)** β-galactosidase assay in TOPGAL chondrocytes (*n* = 3). Ct, Threshold cycle. **(B)** Western blot analysis of nuclear proteins showing inhibition of the translocation of β-catenin to the nucleus by sclerostin (Scl) treatment (*n* = 3). **(C)** Sclerostin partially restored the expression of the anabolic markers (*COL2A1*, *ACAN* and *SOX9*) (*n* = 6). **(D)** Wnt3a-inhibited proteoglycan release by chondrocytes was restored with sclerostin treatment (*n* = 6). **(E)** Sclerostin abolished the Wnt3a-increased gene expression of catabolic markers (*ADAMTS4*, *ADAMTS5*, *MMP3* and *MMP13*) (*n* = 5). **(F)** and **(G)** Sclerostin abolished the Wnt3a-increased expression of hypertrophic markers (*COL10A1* and *VEGF* (*n* = 8). **(H)** Western blot analysis of sclerostin-inhibited expression of Adamts-4 and Adamts-5 and type X collagen (*n* = 3). **(I)** Quantification of Alcian Blue staining showing that Wnt3a reduced the accumulation of highly sulfated glycosaminoglycans (GAGs) (*n* = 5). Wild-type (WT) chondrocytes were used for all the experiments. Data are mean ± SEM. *P < 0.05 versus control; £*P* < 0.05 versus Wnt3a.

Wnt3a elicited matrix catabolism and inhibited chondrocyte anabolism in primary articular chondrocytes harvested from human and rabbit knees [27]. Therefore, we investigated whether inhibition of the Wnt/β-catenin pathway by sclerostin-affected chondrocyte metabolism. Using a β-Gal assay with TOPGAL chondrocytes, we confirmed that Wnt3a increased Wnt/β-catenin transcriptional activity by enhancing the translocation of β-catenin into the nucleus (Figure 5A and B). Similar results were obtained with mouse recombinant Wnt3a (Additional file 1: Figure S1B). Furthermore, Wnt3a reduced the expression of anabolic markers (*COL2A1*, *ACAN*, *SOX9*) but enhanced that of catabolic (*ADAMTS5/4*, *MMP3*, *MMP13*) and hypertrophic markers (*COL10A1* and *VEGF*) (Figures 5E to G). In contrast, sclerostin inhibited Wnt/β-catenin transcriptional activity (Figure 5A and B) and abolished the induction of the gene and protein expression of catabolic and hypertrophic markers (Figure 5E to H). Therefore, sclerostin prevented the induction of catabolic markers and the shift toward the hypertrophic phenotype. Moreover, it restored the amount of proteoglycan release (Figure 5D) and rescued in part the gene expression of anabolic markers (Figure 5C).

### Sclerostin restores the Wnt3a-inhibited expression of anabolic markers via JNK

**Figure 6:**
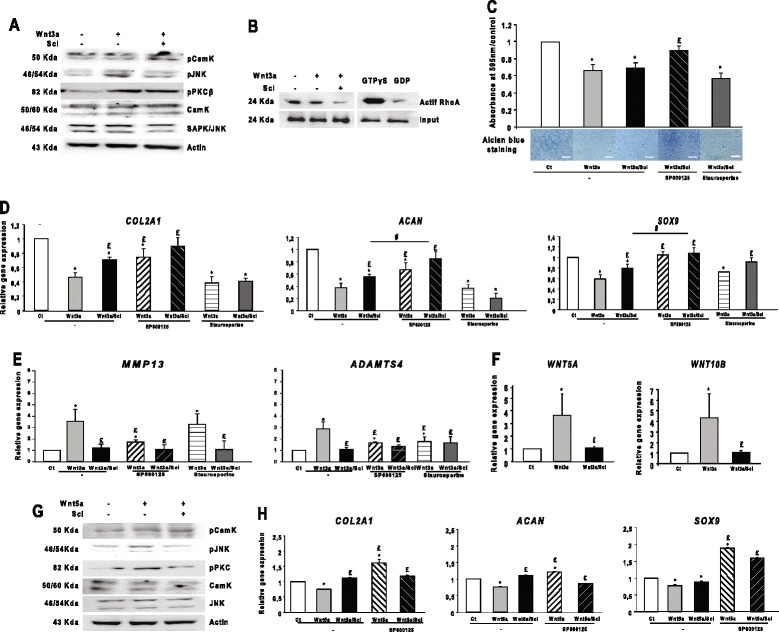
**Sclerostin restores the expression of anabolic markers by inhibiting the c-Jun N-terminal kinase in chondrocytes. (A)** Western blot analysis of the activation of c-Jun N-terminal kinase (JNK) and protein kinase C (PKC) pathways with Wnt3a and sclerostin (*n* = 3). **(B)** Active Rho pull-down and detection (*n* = 3). **(C)** Alcian blue staining and spectrophotometric quantification of highly sulfated glycosaminoglycans (GAGs). Bar, 100 nm (n = 7). **(D)** Sclerostin fully restored the gene expression of anabolic markers with treatment with the JNK inhibitor SP600125, but not the PKC inhibitor staurosporine (*n* = 7). **(E)** Sclerostin alone was sufficient to inhibit the increased gene expression of catabolic markers (*n* = 7). **(F)** Sclerostin inhibited the Wnt3a-increased gene expression of *WNT5A* and *WNT10B* (*n* = 9). **(G)** Western blot analysis of the activation of JNK, CaM kinase (CaMK) and PKC pathways with Wnt3a and sclerostin (*n* = 3). **(H)** Sclerostin rescued Wnt5a-inhibited gene expression of anabolic markers (*n* = 3). Wild-type chondrocytes were used for all the experiments. Data are mean ± SEM. *P < 0.05 versus controls; £*P* < 0.05 versus Wnt5a.

Because anabolic genes might be inhibited by non-canonical Wnt pathways [28], we hypothesized that sclerostin might regulate the anabolic genes via non-canonical Wnt pathways. Wnt3a modulates the articular chondrocyte phenotype by activating both canonical and non-canonical pathways [29]. Indeed, Wnt3a can activate JNK, PKC, Ca^2+^/CaMKII and RhoA [30]. We investigated the Wnt3a-induced activation of JNK, PKC, Ca^2+^/CaMKII and RhoA in chondrocytes. Wnt3a activated JNK and PKC, but not RhoA or Ca^2+^/CaMKII; sclerostin treatment inhibited phosphorylated JNK only (Figure 6A and B). Sclerostin completely restored the production of highly sulfated GAGs and the gene expression of anabolic markers in the presence of JNK inhibitor SP600125, but not the PKC inhibitor staurosporine (Figure 6C and D), thereby restoring the expression of anabolic markers by inhibiting the Wnt3a-induced JNK pathway. Moreover, the expression of the catabolic markers *ADAMTS4* and *MMP13* was not affected by SP600125 or staurosporine (Figure 6E), indicating that their gene expression was regulated mainly by the canonical Wnt pathway.

JNK is activated by non-canonical Wnt5a [31], with its expression upregulated by Wnt3a [32]. Here, Wnt3a increased the expression of *WNT5A* and *WNT10B*, which was abolished by sclerostin (Figure 6F). Furthermore, sclerostin reduced the Wnt5a-induced phosphorylation of JNK and PKC, but it had no effect on the phosphorylation of Ca^2+^/CaMKII (Figure 6G). Wnt5a inhibited the expression of anabolic markers, which was reversed by the JNK inhibitor SP600125 (Figure 6H), thus by activating JNK. Sclerostin restored the gene expression of anabolic markers inhibited by Wnt5a, indicating that it regulated the anabolic markers in chondrocytes via the JNK non-canonical pathway.

## Discussion

We demonstrate here that SOST-KO mice showed severe OA in response to joint instability induced by DMM. Thus, sclerostin plays a role in cartilage maintenance during OA development. OA is associated with early high-level bone remodeling followed by a slow turnover, which leads to bone densification and cartilage loss [26]. We show that SOST deficiency prevented the decrease of BMD observed in WT mice (week 4 of DMM). Thus, sclerostin inhibits bone formation in the subchondral bone in early stages of OA as described in whole skeleton. Moreover, we show that osteophyte volume was not enhanced in DMM SOST-KO mice, suggesting that sclerostin is not involved in osteophyte formation. We found that sclerostin was expressed in the joint cartilage of mice, as previously reported in sheep, mice and humans [15], although it was restricted to calcified cartilage. As already shown in bone, sclerostin is expressed in response to mechanical loading [33]. The high expression in OA cartilage may be an adaptive response to joint instability as a compensatory mechanism to prevent cartilage breakdown. Indeed, SOST-KO mice showed no cartilage lesions in the absence of joint instability despite high bone mass [16]. Thus, high subchondral BV is not sufficient for the development of cartilage lesions, as demonstrated in SOST-KO aged mice and by anti-sclerostin antibodies in OA rats [16]. In our study, joint instability exacerbated cartilage degradation, and we show that sclerostin plays a crucial role in cartilage remodeling when submitted to mechanical stress independently of bone mass. Moreover, we found that sclerostin reduced anabolic markers in primary chondrocytes, unlike previous observations in cartilage explants [15]. This might be related to the matrix environment, which might contribute to the regulation of the expression of matrix genes.

Pharmacological treatment with systemic sclerostin antibody injections in rats treated with MMT had no effect on the onset or severity of OA, despite an increased BV/TV [16] that could suggest that the production of sclerostin is not necessary to protect against cartilage injuries. However, the absence of inhibition by the antibodies might be related to the poor diffusion of antibodies into the cartilage. Therefore, our observations in full *SOST*-KO mice are the effect of sclerostin deletion in both bone cells and hypertrophic chondrocytes. Taken as a whole, these findings suggest that sclerostin produced by chondrocytes is necessary to prevent cartilage lesions under conditions of mechanical stress. Adamts4 and Adamts5 are two key proteases involved in cartilage degradation in OA. Cartilage lesions are associated with increased expression of Adamts4 and Adamts5 that indicate that sclerostin has anticatabolic properties.

We found that lack of sclerostin increased cell apoptosis independently of OA induction in the non-calcified and calcified cartilage. These data suggest that sclerostin inhibits chondrocyte apoptosis physiologically. Nevertheless, DMM exacerbated cell death in SOST-KO mice, indicating an additive effect of SOST deletion and mechanical joint instability in chondrocyte apoptosis. Some evidence has shown that excess Wnt signaling promotes cartilage breakdown and OA [7,8]. Here we found that SOST deficiency resulted in increased OA score. Our findings also confirmed that catabolic activity in chondrocytes is enhanced by overactivation of the Wnt/β-catenin pathway with SOST deficiency, as was previously reported in several i*n vitro* studies [27,29,31]. Characterization of Wnt pathways in cartilage metabolism is still a matter of debate, because both overactivation and inhibition of Wnt/β-catenin signaling results in increased cartilage damage [34,35]. Wnt3a activates both canonical and non-canonical Wnt pathways in chondrocytes, but little is known about the role of sclerostin in regulating these Wnt pathways and chondrocyte metabolism. We show here that sclerostin maintained chondrocyte homeostasis by several mechanisms, such as by reducing the expression of catabolic markers and chondrocyte apoptosis induced by Wnt3a and restoring the expression of anabolic genes. In analyzing non-canonical Wnt signaling, Wnt3a activated the chondrocytic JNK and PKC pathways, but only JNK restored the expression of type II collagen and aggrecan. Sclerostin alone partially rescued the anabolic activity in chondrocytes, and the additional blockade of JNK by sclerostin and SP60025 fully rescued the expression of these anabolic markers. Therefore, we believe that sclerostin is necessary to restore full anabolic activity in chondrocytes, with its action being mediated at least in part by inhibition of the JNK pathway which specific forms remains to be demonstrated.

We failed to observe any activation of Ca^2+^/CaMKII signaling, in contrast to a previous report in human osteoarthritic chondrocytes [29]. Thus, distinct signaling pathways could be involved in damaged and undamaged chondrocytes. However, our work provides further evidence of a crosstalk between Wnt/β-catenin and JNK signaling in regulating anabolic activity in chondrocytes, which suggests a cooperative effect between these two pathways. Moreover, sclerostin was able to inhibit the expression of *WNT5A* induced by the activation of β-catenin, and the inhibition of JNK by sclerostin may be related to the inhibition of the β-catenin-dependent pathway, thus resulting in a lower expression of the *WNT5A* gene.

Our study provides new insights into the role of sclerostin in the onset and progression of OA. Sclerostin was necessary to protect cartilage against lesions produced by mechanical joint instability in mice. Because of dual action of sclerostin in the canonical and non-canonical Wnt pathways, it is an interesting target in attempting to preserve chondrocyte metabolism. Finally, if complete deletion of SOST might be deleterious to cartilage, attention needs to be paid to the joints of patients with osteoporosis who may require anti-sclerostin antibody therapy.

## Conclusions

We demonstrate that lack of sclerostin aggravated the development of OA in mice submitted to joint instability as a result of disrupted anabolic/catabolic balance. Moreover, sclerostin acts through the inhibition of the canonical Wnt pathway as well as through the inhibition of the non-canonical JNK pathways in chondrocytes, thus preserving metabolism. Indeed, sclerostin may play an important role in maintaining cartilage integrity under mechanical stress.

## Additional file

Additional file 1: Figure S1. Wnt3a conditioned media enhances Wnt/β-catenin signaling in a dose- and time-dependent manner. (A) β-galactosidase activity measurement in TOPGAL chondrocyte cultures induced by 10% or 30% Wnt3a conditioned media (cmWnt3a) (*n* = 5). **(B)** β-galactosidase activity measurement in TOPGAL chondrocyte cultures induced by 40 ng/ml recombinant mouse Wnt3a (*n* = 3). **(C)** β-galactosidase activity measurement in TOPGAL chondrocyte cultures induced for 48 hours or 96 hours by 30% cmWnt3a (*n* = 3). Data are mean ± SEM. **P* < 0.05 versus control, #*P* < 0.05.

## Abbreviations

ACAN: Aggrecan
ADAMTS: A disintegrin and metalloproteinase with thrombospondin motifs (aggrecanase)
BMD: Bone mineral density
BV/TV: Bone volume/tissue volume
CaMKII: CaM kinase II
C_t_: Threshold cycle
(DMM: Destabilization of the medial meniscus
GAG: Glycosaminoglycan
β-Gal: β-galactosidase
IL-1β: Interleukin 1β
JNK: c-Jun N-terminal kinase
MMP: Metalloproteinase
MMT: Medial meniscus transection
OA: Osteoarthritis
OCT: Optimum cutting temperature
PKC: Protein kinase C
RhoA: Ras homolog member A
SAPK: Stress-activated protein kinase
SOX9: SRY (sex determining region Y)-box 9
TUNEL: Terminal deoxynucleotidyl transferase dUTP nick end labeling
Wnt: Wingless-related integration site
WT: Wild type
