# Supplementary figures and images for: Loss of sclerostin promotes osteoarthritis in mice via β-catenin-dependent and -independent Wnt pathways
Source: Arthritis Res Ther. 2015 Feb 6;17(1):24. doi: 10.1186/s13075-015-0540-6 (PMC4355467; doi:10.1186/s13075-015-0540-6)

**A**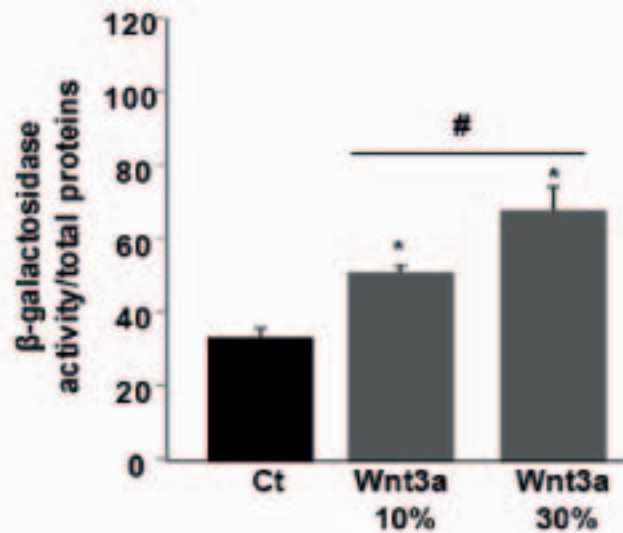**B**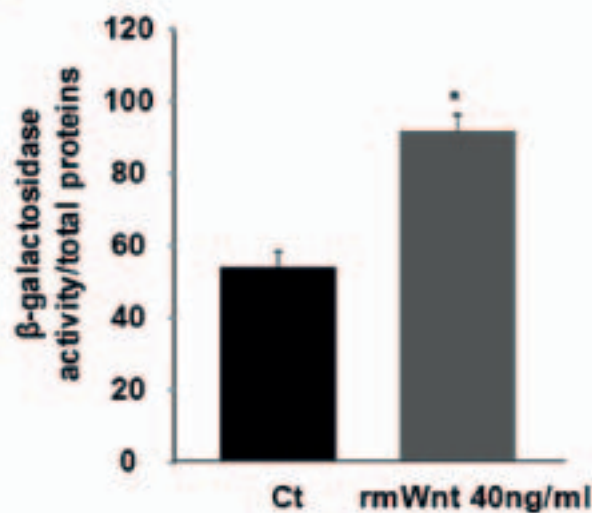**C**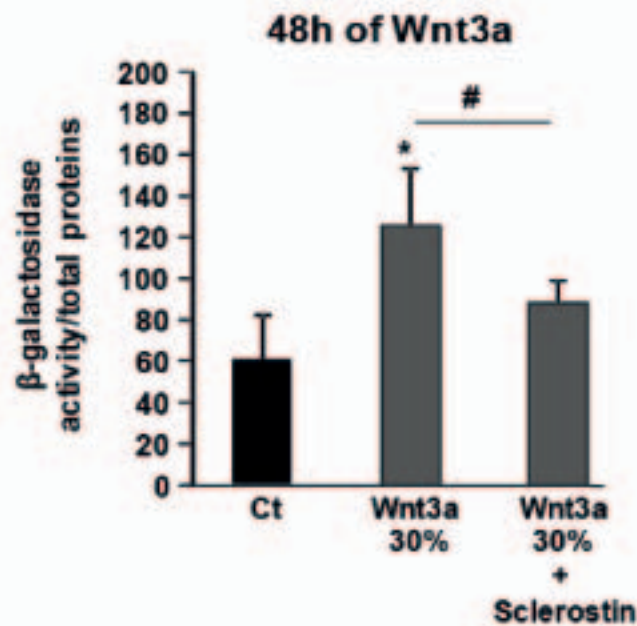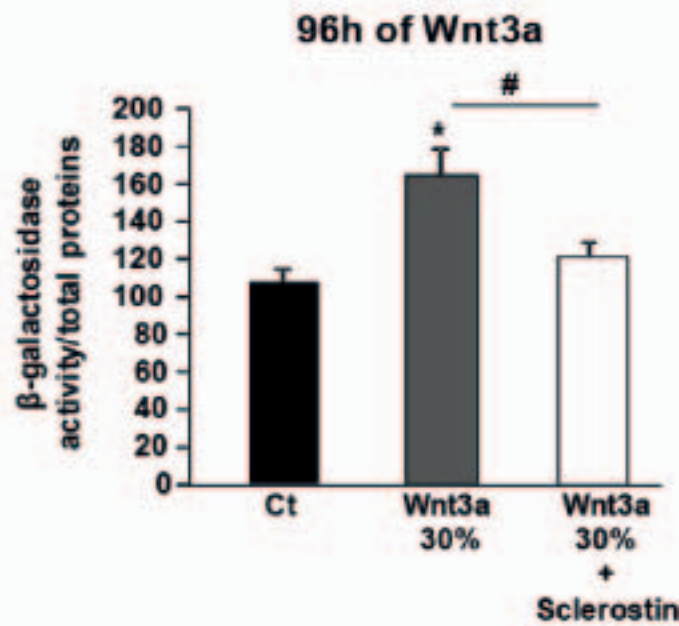

Supplement: Additional file 1: Figure S1. — Wnt3a conditioned media enhances Wnt/β-catenin signaling in a dose- and time-dependent manner. (A) β-galactosidase activity measurement in TOPGAL chondrocyte cultures induced by 10% or 30% Wnt3a conditioned media (cmWnt3a) (n = 5). (B) β-galactosidase activity measurement in TOPGAL chondrocyte cultures induced by 40 ng/ml recombinant mouse Wnt3a (n = 3). (C) β-galactosidase activity measurement in TOPGAL chondrocyte cultures induced for 48 hours or 96 hours by 30% cmWnt3a (n = 3). Data are mean ± SEM. *P < 0.05 versus control, #P < 0.05. [file 13075_2015_540_MOESM1_ESM.pdf]
